# FTLD‐TDP‐43 With Motor Neuron Disease Pathology in an Autopsied Patient With Spastic Paraplegia‐30B Harbouring a Homozygous *KIF1A* Variant

**DOI:** 10.1111/nan.70079

**Published:** 2026-05-13

**Authors:** Rie Saito, Arika Hasegawa, Tetsuya Takahashi, Ryoko Koike, Norikazu Hara, Ramil Gabdulkhaev, Kishin Koh, Akio Kawakami, Yoshihisa Takiyama, Takeshi Ikeuchi, Akiyoshi Kakita

**Affiliations:** ^1^ Department of Pathology, Brain Research Institute Niigata University Chuo‐ku Niigata Japan; ^2^ Center for Human Brain Resource Initiative (ChBRI) Niigata University Chuo‐ku Niigata Japan; ^3^ Department of Neurology NHO Nishiniigata Chuo Hospital Nishi‐ku Niigata Japan; ^4^ Department of Molecular Genetics Brain Research Institute, Niigata University Chuo‐ku Niigata Japan; ^5^ Department of Neurology Yumura Onsen Hospital Kofu Yamanashi Japan; ^6^ Department of Neurology Kaetsu Hospital Akiha‐ku Niigata Japan; ^7^ Department of Neurology, Graduate School of Medical Sciences University of Yamanashi Chuo Yamanashi Japan; ^8^ Department of Neurology Fuefuki Central Hospital Fuefuki Yamanashi Japan

**Keywords:** FTLD‐TDP‐43 with MND, hereditary spastic paraplegia type 30B, *KIF1A* variant, neuropathology, SCA31

## Abstract

- We report for the first time the presence of FTLD‐TDP‐43 with MND pathology in an autopsied patient with childhood‐onset hereditary spastic paraplegia (HSP) due to a homozygous *KIF1A* variant.
- Clinicopathological findings suggested that FTLD‐TDP‐43 with MND pathology had developed at the end of the disease course.
- The present case highlights the clinicopathological heterogeneity of *KIF1A*‐associated neurological disorder and suggests that HSP caused by *KIF1A* variants may share a pathological continuum with FTLD and ALS through a TDP‐43–related pathway.

## Graphical abstract

*KIF1A*‐associated neurological disorder (KAND) is a rare hereditary condition caused by KIF1A variants, affecting axonal transport and presenting with a wide clinical spectrum, including hereditary spastic paraplegia. This case of childhood‐onset KAND reveals FTLD‐TDP43 with motor neuron disease pathology emerging late in the disease course, suggesting that HSP and FTLD‐MND share a pathological continuum through a TDP‐43‐related pathway and expanding the clinicopathological spectrum of KAND.

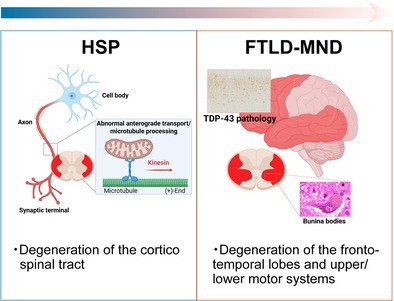

**Abbreviations**
FTD: frontotemporal dementia
FTLD‐TDP‐43 with MND: frontotemporal lobar degeneration‐TDP‐43 with motor neuron disease
GCIs: glial cytoplasmic inclusions
HSP‐30B: hereditary spastic paraplegia type 30B
KAND: *KIF1A*‐associated neurological disorder
NCIs: neuronal cytoplasmic inclusions
SCA31: spinocerebellar ataxia type 31

*KIF1A* encodes a neuron‐specific kinesin‐3 motor protein that mediates the anterograde axonal transport of synaptic vesicle precursors, an essential process for synaptic maintenance and neuronal survival [1]. Variants in *KIF1A* are thought to disrupt axonal transport dynamics, leading to progressive neurodegeneration. Initial reports identified homozygous *KIF1A* variants in hereditary sensory and autonomic neuropathy type II [2] and in hereditary spastic paraplegia type 30B (HSP‐30B/SPG30B), an autosomal recessive spastic paraplegia characterised by slowly progressive lower limb spasticity due to corticospinal tract degeneration [3]. As accumulated reports have now led to the recognition that *KIF1A*‐associated neurological disorder (KAND) encompasses a broad clinical spectrum including neuropathy, cerebellar ataxia, intellectual disability, cortical visual impairment, and severe encephalopathy [4]; it has also been recognised that a subset of patients diagnosed as having sporadic amyotrophic lateral sclerosis (ALS) harbour *KIF1A* variants [5], further emphasising the extent of motor system involvement within KAND. Despite this expansion of the clinical spectrum, the neuropathological features remain largely unknown. Here, we report the neuropathological features of a Japanese male with HSP‐30B harbouring a homozygous *KIF1A* variant.

The clinical data for this patient have been reported previously in detail [6]. Briefly, the patient came from a consanguineous family in which several relatives had been genetically confirmed to have spinocerebellar ataxia type 31 (SCA31). At the age of five, the patient developed spasticity with intellectual disability followed by cerebellar ataxia, and became wheelchair‐bound in his mid‐50s. Genetic analysis revealed coexisting HSP‐30B and SCA31, harbouring a homozygous *KIF1A* variant (c.726C > G, p.Ser242Arg) and an approximately 2.7‐kb insertion corresponding to ~760 TGGAA repeats in the *BEAN* and *TK2* genes (Figure S1). Repeat‐primed PCR for the *C9orf72* GGGGCC hexanucleotide repeat expansion revealed no pathological expansion. Whole‐exome sequencing‐based screening for ALS, FTLD, and motor neuron disease–related genes (*SOD1*, *FUS*, *TARDBP*, *SETX*, *VAPB*, *ANG*, *FIG4*, *OPTN*, *ATXN2*, *DAO*, *C9orf72*, *ALS2*, *SIGMAR1*, *UBQLN2*, *CHMP2B*, *VCP*, *MATR3*, *NEFH*, *NEFL*, *SQSTM1*, *TAF15*, *ERBB4*, *CHCHD10*, *TBK1*, *GLE1*, *PFN1*, *TUBA4A*, *HNRNPA1* and *HNRNPA2*) within the Japan Spastic Paraplegia Research Consortium panel identified no pathogenic variants. He presented with time disorientation and dysphagia without apparent muscle weakness, and subsequently died of asphyxia due to food aspiration at the age of 65. A general autopsy was performed, at which time the brain weighed 1150 g (cerebrum 1000 g; cerebellum and brainstem 150 g). Annual brain MRI examinations from ages 51 to 56 showed mild cerebral and cerebellar atrophy without progression, and no features suggestive of a frontotemporal dementia (FTD) pattern (Figure S2).

Macroscopically, diffuse cerebral atrophy was evident, being most pronounced in the frontal and anterior temporal lobes and motor cortex (Figure 1A). The motor cortex showed moderate loss of Betz cells on the medial side, whereas degeneration was more pronounced on the lateral side. In these regions, mild to moderate neuronal loss with gliosis was accompanied by phosphorylated TDP‐43 pathology, including neuronal and glial cytoplasmic inclusions (NCIs and GCIs), the burden of which increased in parallel with the extent of neuronal loss (Figure 1B–D). The NCIs were evident as granular to small dot‐like structures, accompanied by some short neurites and numerous TDP‐43‐immunoreactive threads, the latter being present across all cortical layers but comparatively fewer in layer IV (Figure 1D,E); p62 immunohistochemistry demonstrated granular positivity in a proportion of NCIs, as well as labelling of GCIs and dystrophic neurites (Figure S3). In addition, some NCIs were observed in the dentate granule cells, without lentiform neuronal intranuclear inclusions (Figure 1F). GCIs were fewer in number than NCIs in the cortex and rare in the white matter and corpus callosum (Figure 1G and Figure S3). In contrast, involvement of lower motor neurons was relatively mild. In the spinal cord, myelin pallor was evident in the lateral and anterior funiculi (Figure 1H, black arrows). Anterior horn cells exhibited mild‐to‐moderate neuronal loss with Bunina bodies (Figure 1I–K), as well as scattered granular NCIs, while only a few GCIs were identified (Figure 1L). Interestingly, although the cervical anterior roots were thin, their myelinated fibres were largely preserved, and regeneration clusters were also evident (Figure 1M). The lower motor neurons in the brainstem, including the hypoglossal nuclei, showed only mild involvement (Figure 1N). These histopathological findings were consistent with frontotemporal lobar degeneration‐TDP‐43 with motor neuron disease (FTLD‐TDP‐43 with MND). Despite the abundant accumulation of TDP‐43, the degree of neuronal loss and cortical atrophy was disproportionately mild. Moreover, the feature could not be assigned to any of the established subtypes (A–E) [7]. In the sensory nervous system, the gracile funiculus exhibited mild pallor, and the dorsal root ganglia showed moderate loss of ganglion cells with Nageotte nodules (Figure 1O), while myelinated posterior roots were preserved (Figure S4). Table 1 summarises the neuronal loss and distribution of the TDP‐43 inclusions.

**FIGURE 1:**
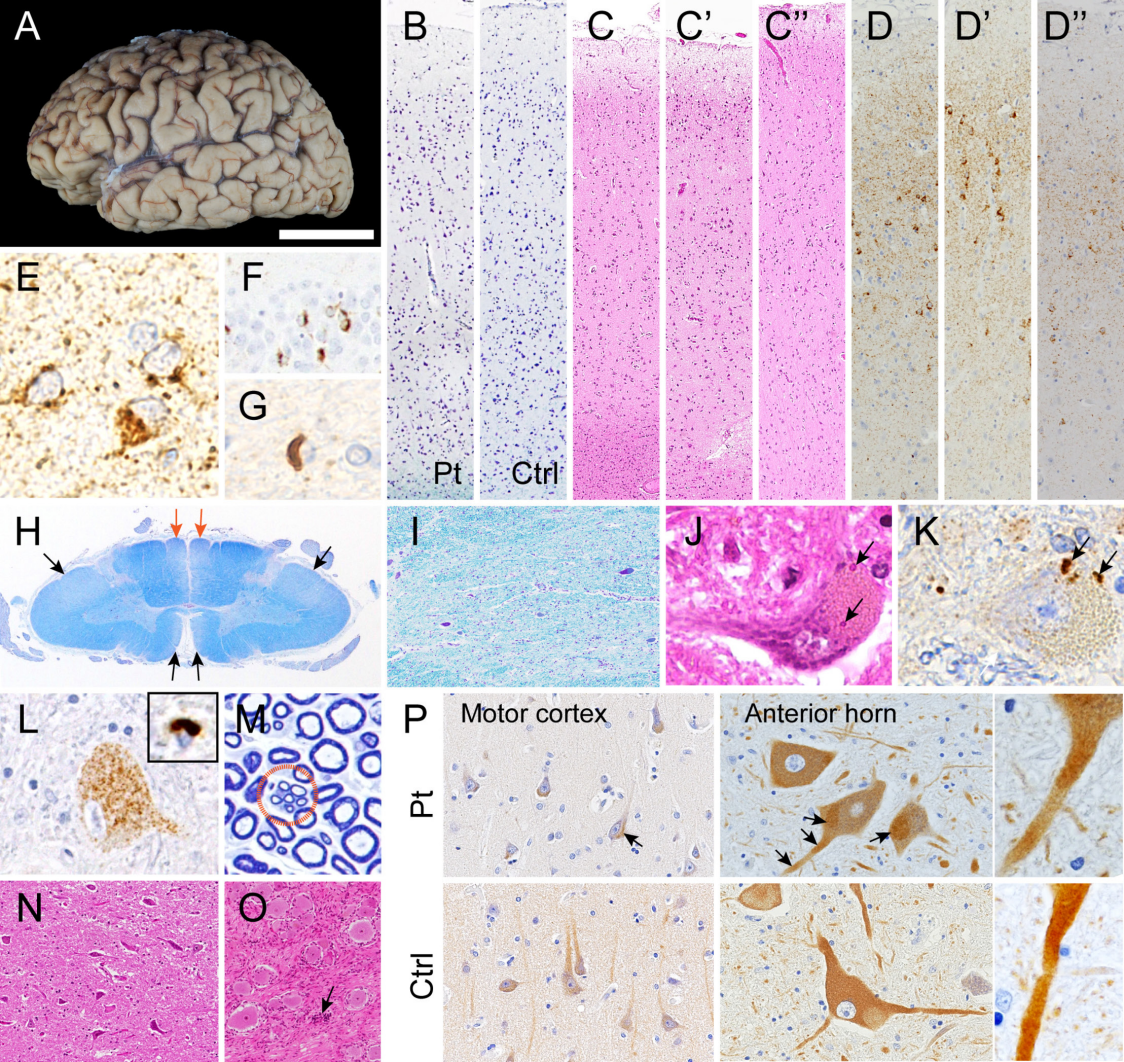
Neuropathological findings. (A) Fixed cerebrum showing frontotemporal atrophy. (B, C) Neuronal loss with gliosis in the cortex: moderate in the frontal cortex (B, C), entorhinal cortex (C′), and motor cortex (C″). (B) Klüver–Barrera staining (KB). (C) H&E staining (HE). (D) Abundant accumulation of phosphorylated TDP‐43 (pTDP‐43) with many neuronal cytoplasmic inclusions (NCIs) and threads in the frontal (D), entorhinal (D′) and motor cortices (D″). (E) NCIs possessing small paranuclear granular deposits in the motor cortex, (F) crescent‐shaped NCIs in the hippocampal dentate granule cells, and (G) a glial cytoplasmic inclusion (GCI), all labelled with pTDP‐43. (H) Myelin pallor of the lateral and anterior funiculi (black arrows), and the gracile fasciculus (orange arrows; KB). (I) Moderate neuronal loss in the cervical anterior horn cells. (J) Bunina bodies in an anterior horn cell. (K) detected with cystatin C. (L) NCIs showing granular deposits in an anterior horn cell, and a GCI (inset). pTDP‐43. (M) Retained myelinated fibres with regeneration clusters of small, thinly myelinated grouped fibres (circle) in the cervical spinal roots. (N) Mild neuronal loss with gliosis in the hypoglossal nucleus (HE). (O) Shrinkage of ganglion cells with a Nageotte nodule (arrow) in the dorsal root ganglion of the lumbar spinal cord. Toluidine blue staining. (P) KIF1A immunohistochemical findings. Note KIF1A has aggregated in the patient's neuronal somata and proximal processes (arrows); right panels show higher magnification of neurites. Pt, patient; Ctrl, control; Neuronal ab, pan‐neuronal marker. Bar in A = 4 cm for A; 400 μm for B, C, I; 200 μm for D; 30 μm for E, F; 90 μm for G; 3 mm for H; 40 μm for J, K, M; 50 μm for L; 350 μm for N; 250 μm for O; and 110 μm (left panel) and 75 μm (right panel) for P.

**TABLE 1:** Summary of neuropathological findings.

	Neuronal loss and gliosis	Density of TDP‐43‐ir inclusions		
		Neurons	Glia	Threads
Cerebral cortex				
Frontal	1–2	3	3	3
Premotor/motor	1/1 (medial)–2 (lateral)	2/3	1/3	1/2
Cerebral white matter	1^a^	NA	1	NA
Parietal	1	1	1	1
Temporal	1	3	2	2
Transentorhinal	0.5	3	1	1
Occipital	1	1	1	1
Subcortical areas				
Hippocampus^b^/dentate gyrus	1–2/0	3/1	3/0	2/1
Amygdala	2^c^	3	1	2
Caudate/putamen/GP i/e	1/1/1/1	1/1/1/3	2/1/3/2	3/0/2/2
Internal capsule	0	NA	1	0
Thalamus	1	1	1	0
Subthalamic nucleus	0	0	1	0
Brain stem				
Periaqueductal grey	1	1	1	1
Red nucleus	0	2	2	1
Substantia nigra	0	2	1	2
V/VII/XII	0/1^d^/1	1/1/1	0/1/1	0/0/0
Pontine nucleus	0	1	1	0
Fibres in pontine base	0	NA	1	0
IO (dorsal/ventral)	1/0	0/1	0/1	0/1
Cerebellum				
Cerebellar cortex/wm	2/1	0/0	0/0	0/0
Dentate nucleus	1	0	0	0
Spinal cord				
Anterior horn C/T/L	2/1/1^d^	2/1/1	1/1/1	0/0/0
Clarke's column	0	1	0	0
Posterior horn	0	1	0	0
IML	0	0	0	0
Corticospinal tract	2^a^	NA	1	0
Posterior column	1^a^	NA	0	0

*Note:* Neuronal loss and gliosis were evaluated semiquantitatively in HE, KB and GFAP‐immunostained sections by comparison with age‐matched control brains (60–70 years): 0, none; 1, mild; 2, moderate; 3, severe. Density of TDP‐43‐immunoreactive (TDP‐43‐ir) inclusions was graded as follows: Neurons—0, 0%; 1, < 25%; 2, 25%–50%; 3, > 50%. Glia—0, 0 per ×20 field; 1, < 10; 2, 10–19; 3, ≥ 20. Threads—0, none; 1, mild; 2, moderate; 3, frequent. GP i/e, globus pallidus (internal/external segments); V, trigeminal nerve nucleus; VII, facial nucleus; XII, hypoglossal nucleus; IO, inferior olivary nucleus; WM, white matter; C, C7 segment of the cervical spinal cord; T, Th8 segment of the thoracic spinal cord; L, L4 segment of the lumbar spinal cord; IML, intermediolateral nucleus.

^a^ Myelin pallor.

^b^ CA1–subiculum region, mild neuronal loss with gliosis in the left anterior and right regions, and moderate neuronal loss with gliosis in the left posterior region.

^c^ Coexisting argyrophilic grain disease (Saito stage III).

^d^ Bunina bodies were present.

To investigate KIF1A alteration in the affected brain and spinal cord, we performed immunohistochemistry using an antibody against C‐terminal KIF1A. In controls, KIF1A immunoreactivity was distributed diffusely throughout neuronal somata and processes, whereas in the patient, it appeared aggregated within the somata and proximal processes of both cortical and spinal motor neurons (Figure 1P).

Within the SCA31 pathology spectrum, cerebellar atrophy was moderate, and the cortex showed moderate loss of Purkinje cells with halo‐like amorphous materials, which are characteristic features of SCA31 [8]. Mild neuronal loss with gliosis was also present in the dorsal regions of the inferior olivary nucleus (Figure S5). In terms of age‐related pathology, the brain showed argyrophilic grain disease (Saito stage III), but only sparse Alzheimer's disease neuropathological change (ABC score: A2B1C1) and no pathological features of Parkinson's disease. Details of the methods used for the genetic and neuropathological examinations are provided in the Supporting Information.

We have described the neuropathological features of a patient with the *KIF1A*‐associated spastic paraplegia phenotype, and unexpectedly identified evidence of FTLD‐TDP‐43 with MND. In KAND, reported pathological data are limited, and no studies have documented cases presenting with an ALS phenotype; thus, the association of MND with TDP‐43 pathology has remained unclear.

The only autopsy report to date described two paediatric cases harbouring a heterozygous c.296C > T variant, both of whom manifested intellectual disability, seizure and spasticity, and exhibited a consistent pattern of severe degeneration affecting the cerebellum, dentate nucleus and inferior olivary nucleus [9]. These features differ from those of the present patient, in whom the cerebellar cortex showed moderate involvement with mild changes in the inferior olivary nucleus. As heterozygous KAND cases are known to have a more severe phenotype than homozygous cases [3]—the latter being the group to which the present patient belonged—the relatively mild cerebellar degeneration observed here was consistent with the homozygous form and therefore more likely attributable to SCA31, with only a minor contribution from the *KIF1A* variant.

Although uncommon, this patient also harboured SCA31, with a *BEAN1*/*TK2* repeat expansion of approximately 2.7 kb, within the reported pathogenic range (2.5–3.8 kb) [10]. Importantly, family members carrying the expansion in the absence of the homozygous *KIF1A* variant exhibited only cerebellar ataxia without spastic paraplegia, whereas spastic paraplegia was observed exclusively in this patient harbouring both genetic abnormalities (Figure S1). These findings indicate that the *BEAN1*/*TK2* expansion accounted for the pure cerebellar phenotype, while the spastic paraplegia was attributable to the *KIF1A* variant. Furthermore, the neuropathological features of the cerebellar system were consistent with other SCA31 cases, and in fact relatively mild for the disease duration, the degeneration being confined to the cerebellar cortex, lacking any evident association with TDP‐43 proteinopathy. Thus, the two conditions would most likely be clinically and pathologically unrelated.

The absence of frontotemporal atrophy on MRI up to age 56, the emergence of frontotemporal and bulbar symptoms only in the final year and the mild lower motor neuron involvement on pathological examination together indicate that the FTLD‐MND developed at the terminal stage of the disease, suggesting that the childhood‐onset spastic paraplegia had been unrelated to FTLD‐MND with TDP‐43 pathology. This raises the issue of the pathology that may have been responsible for the spastic paraplegia. A previous study of another form of HSP caused by *SPAST* variants demonstrated pallor of the corticospinal tracts—most prominently in the thoracic and lumbar cord—consistent with a distal ‘dying‐back’ axonopathy of long tracts [11]. In the present case, pallor was also noted in the lateral columns of the spinal cord; however, no clear distal predominance was evident, implying that the changes in the corticospinal tract may have reflected degeneration associated with FTLD‐MND rather than structural alterations underlying spastic paraplegia; any contribution from the latter, if present, would likely have been minimal. In *KIF1A*‐deficient mice, the absence of this kinesin‐3 motor protein has been shown to impair axonal transport of synaptic vesicle precursors, leading to reduced synaptophysin immunoreactivity at synaptic terminals and vesicular accumulation in neuronal somata [12]. Subsequent studies have demonstrated that pathogenic *KIF1A* variants can instead enhance KIF1A motility, resulting in distal aggregation of KIF1A‐positive puncta along axons [13], while iPSC‐derived motor neuron models have revealed proximal accumulation of KIF1A with autophagic impairment and neurite degeneration [14]. Together, these features indicate that both loss and gain of KIF1A function disrupt the homeostasis of axonal transport, leading to spastic paraplegia. In the present patient, the homozygous loss‐of‐function variant and KIF1A aggregation within neuronal somata and processes, consistent with previous reports, would suggest a functional disturbance of axonal transport, even in the absence of apparent morphological alterations.

Among established FTLD‐TDP subtypes, type E most closely resembles the present case, sharing granular NCIs distributed across cortical layers [7, 15]. However, several features differ. In the original type E series, neuronal granulofilamentous neuronal inclusions and grains were largely p62‐negative, with < 5% showing moderate immunoreactivity, whereas oligodendroglial inclusions consistently showed strong p62 positivity. In contrast, in the present case, a proportion of NCIs showed granular p62 positivity, and similar labelling was also observed in GCIs and dystrophic neurites, indicating broader p62 immunoreactivity than seen in type E neuronal inclusions. Furthermore, although GCIs were present in both grey and white matter, they were represented only sparsely in the white matter and corpus callosum, where they are frequently seen in type E. In the present case, Western blot analysis to assess the ~26 kDa C‐terminal fragment predominance of type E was not performed, and was therefore a limitation of this study.

It remains unknown whether *KIF1A* variants cause cytoplasmic mislocalisation of TDP‐43. On the other hand, variants in the related kinesin gene *KIF5A*, leading to similar disruption of axonal transport, have been shown to drive aggregation of mutant protein and cytoplasmic mislocalisation of TDP‐43 in cellular models [16]. Similarly, autopsied patients with *KIF5A* splice‐site variants exhibit ALS‐TDP‐43 pathology. Moreover, FTLD‐MND with TDP‐43 pathology has also been reported in advanced‐stage HSP due to *NIPA1* variants (SPG6) [17], as in the present patient. These findings suggest that a similar axonal‐transport–related mechanism may have contributed to the development of TDP‐43 pathology in the present case, implying that HSP and FTLD‐MND may lie on a continuous genetic and pathological spectrum.

The present case highlights the clinicopathological heterogeneity of KAND and suggests that HSP caused by *KIF1A* variants may share a pathological continuum with FTLD and ALS through a TDP‐43‐related pathway. Further molecular studies and accumulation of autopsy cases of KAND will be essential for the clarification of how *KIF1A* dysfunction impacts the long tract, including the motor system and disease manifestations.

## Author Contributions

R. Saito and A. Kakita designed the research project and performed the pathological analyses. A. Hasegawa, T. Takahashi, R. Koike and A. Kawakami collected the clinical data. R. Gabdulkhaev prepared the autopsy samples. K. Koh, N. Hara, Y. Takiyama and T. Ikeuchi performed the genetic analyses. R. Saito, N. Hara, A. Hasegawa and A. Kakita drafted the manuscript for intellectual content.

## Funding

This work was supported by the Japan Society for the Promotion of Science (10.13039/501100001691, 24K10617, 23H00434, JPJS00420240016), the Japan Agency for Medical Research and Development (10.13039/100009619, JP23jm0210097, JP24zf0127012) and the Ministry of Health, Labour and Welfare of Japan (JPMH23FC1008, JPMH23FC1010).

## Ethics Statement

The present study was approved by the Ethics Committee of Niigata University (G2019‐0016). Written‐informed consent for autopsy, including the use of tissues for research purposes, genetic investigation and publication was obtained from the patients' families.

## Conflicts of Interest

The authors declare no conflicts of interest.

## Supporting information


**Table S1:** Primary antibodies.
**Figure S1:** Pedigree and genetic analyses demonstrating coexisting HSP‐30B and SCA31 in the family.
**Figure S2:** Brain MRI and CT images.
**Figure S3:** p62 and phosphorylated TDP‐43 immunoreactivity in the frontal lobe and corpus callosum.
**Figure S4:** Pathological findings in the spinal roots and sural nerve.
**Figure S5:** Cerebellar pathology.

## Data Availability

The datasets used and analysed during the present study are available from the corresponding author on reasonable request.
