# Supplementary material for: FTLD‐TDP‐43 With Motor Neuron Disease Pathology in an Autopsied Patient With Spastic Paraplegia‐30B Harbouring a Homozygous KIF1A Variant
Source: Neuropathol Appl Neurobiol. 2026 May 13;52:e70079. doi: 10.1111/nan.70079 (PMC13172651; doi:10.1111/nan.70079)
Supplement: Supplementary file 1 — Table S1: Primary antibodies. Figure S1: Pedigree and genetic analyses demonstrating coexisting HSP‐30B and SCA31 in the family. Figure S2: Brain MRI and CT images. Figure S3: p62 and phosphorylated TDP‐43 immunoreactivity in the frontal lobe and corpus callosum. Figure S4: Pathological findings in the spinal roots and sural nerve. Figure S5: Cerebellar pathology. [file NAN-52-e70079-s001.docx]

**Supplementary File**

***Neuropathology and Applied Neurobiology***

*SHORT COMMUNICATION*

**FTLD-TDP-43 with Motor Neuron Disease Pathology in an Autopsied Patient with Spastic Paraplegia-30B Harbouring a Homozygous *KIF1A* Variant**

Rie Saito^1,2^, Arika Hasegawa^3^, Tetsuya Takahashi^3^, Ryoko Koike^3^, Norikazu Hara^4^, Ramil Gabdulkhaev^1^, Kishin Koh^5^, Akio Kawakami^6^, Yoshihisa Takiyama^7,8^, Takeshi Ikeuchi^4^, Akiyoshi Kakita^1,2^

Departments of Pathology^1^ and Molecular Genetics^4^, Brain Research Institute, Niigata University, Chuo-ku, Niigata, Japan; ^2^Center for Human Brain Resource Initiative (ChBRI), Niigata University, Chuo-ku, Niigata, Japan.; ^3^Department of Neurology, NHO Nishiniigata Chuo Hospital, Nishi-ku, Niigata, Japan; ^5^Department of Neurology, Yumura Onsen Hospital, Kofu, Yamanashi, Japan; ^6^Department of Neurology, Kaetsu Hospital, Akiha-ku, Niigata, Japan; ^7^Department of Neurology, Graduate School of Medical Sciences, University of Yamanashi, Chuo, Yamanashi, Japan; and ^8^Department of Neurology, Fuefuki Central Hospital, Fuefuki, Yamanashi, Japan

**Corresponding Author**:

Rie Saito, MD, PhD

Department of Pathology, Brain Research Institute, Niigata University.

Center for Human Brain Resource Initiative (ChBRI), Niigata University.

1-754 Asahimachi, Chuo-ku, Niigata 951-8122, Japan.

Tel: +81-25-227-0640

e-mail: riesaito@bri.niigata-u.ac.jp

**Supplementary methods**

**Table S1:** Primary antibodies

**Figure S1:** Pedigree and genetic analyses demonstrating coexisting HSP-30B and SCA31 in the family

**Figure S2:** Brain MRI and CT images

**Figure S3:** p62 and phosphorylated TDP-43 immunoreactivity in the frontal lobe and corpus callosum.

**Figure S4:** Pathological findings in the spinal roots and sural nerve

**Figure S5:** Cerebellar pathology

**Supplementary references**

**Supplementary methods**

***Histopathological analysis***

The brain and spinal cord were fixed with 10% buffered formalin, and multiple tissue blocks were embedded in paraffin. Histological examination was performed on 4-μm-thick sections using haematoxylin and eosin and Klüver-Barrera staining. In addition, selected sections were immunostained with antibodies against phosphorylated TDP-43, p62, KIF1A, cystatin C and calbindin D-28k (**Table S1**). Antibodies against amyloid β 11-28, phosphorylated tau and phosphorylated α-synuclein were used to assess senile pathological changes based on the “ABC” score [1] and the fourth consensus report of the DLB Consortium [2]. Argyrophilic grain disease was assessed using Gallyas–Braak silver staining and an antibody against phosphorylated tau [3]. Bound antibodies were visualised by the peroxidase-polymer-based method using a Histofine Simple Stain MAX-PO kit (Nichirei, Tokyo, Japan) with diaminobenzidine as the chromogen. Immunostained sections were counterstained with haematoxylin.

To assess the posterior and anterior spinal roots and sural nerve, tissue was fixed in 2.5% glutaraldehyde in 0.125 M cacodylate buffer and embedded in epoxy resin. Semithin sections were then prepared and stained with toluidine blue.

***Genetic analysis***

The missense p.S242R (c.726C>G) mutation of *KIF1A* and abnormal expansion in *BEAN* and *TK2* were examined by PCR, as reported previously [4]. For detection of repeat expansions, PCR amplification was performed in 10 μL of a reaction mixture containing 10 ng of template DNA, 5 μL of 2× GC Buffer I (TaKaRa Bio Inc., Shiga, Japan), 250 μM each dNTP, 0.5 μM each primer, and 0.5 U of LA Taq polymerase (TaKaRa Bio Inc.). The primers used were 5′-ACTCCAACTGGGATGCAGTTTCTCAAT-3′ and 5′-CTTTAGGGACCTGATTTCCTTCCTCCA-3′. PCR was carried out using a thermal cycler under the following conditions: initial denaturation at 94 °C for 1 min; 33 cycles of denaturation at 94 °C for 20 s and annealing/extension at 64 °C for 8 min; followed by a final extension at 72 °C for 10 min.

PCR products were analysed on the 4200 TapeStation system (Agilent Technologies, Santa Clara, CA, USA) using Genomic DNA ScreenTape (Agilent Technologies) to determine amplicon sizes. The region amplified using the above primers corresponds to 1527 bp in the reference sequence (GRCh38), which includes 14 repeats of the pentanucleotide motif AATAA (totalling 70 bp). Therefore, the non-repeat portion of the amplicon is 1457 bp. To estimate the repeat number from the observed amplicon size, we subtracted 1457 bp from the latter and divided the difference by 5 bp, which represents the length of one repeat unit. The resulting value was rounded to the nearest whole number to obtain the estimated repeat count. For example, if the observed size was 1613 bp, the calculation gives 31.2 repeats, which was rounded to 31.

The *C9orf72* repeat length was analysed as described previously [6].

**Table S1.** **Primary antibodies.**

| Antigen (clone) | Antibody species | Source | Dilution | Antigen retrieval |
| --- | --- | --- | --- | --- |
| Immunohistochemistry |  |  |  |  |
| Calbindin-D28k (CB300) | Mouse | Swant, Fribourg, Switzerland | 1:50 | Microwave |
| Cystatin C | Rabbit | Dako, Glostrup, Denmark | 1:2000 | None |
| KIF1A | Rabbit | Abcam, Cambridge, UK | 1:1000 | None |
| Phosphorylated TDP-43 (pS409/410) | Mouse | Cosmo Bio, Tokyo, Japan | 1:5000 | Autoclave |
| Amyloid β (11-28, 12B2) | Mouse | IBL, Gunma, Japan | 1:50 | Formic acid |
| Phosphorylated tau (AT8) | Mouse | Fujirebio, Ghent, Belgium | 1:200 | None |
| Phosphorylated α-synuclein (pSyn#64) | Mouse | Wako, Saitama, Japan | 1:1000 | Formic acid |
| p62 (3/p62lck) | Mouse | BD Transduction Laboratories™, San Jose, CA, USA | 1:1000 | Microwave |

**Figure S1. Pedigree and genetic analyses demonstrating coexisting HSP-30B and SCA31 in the family.**


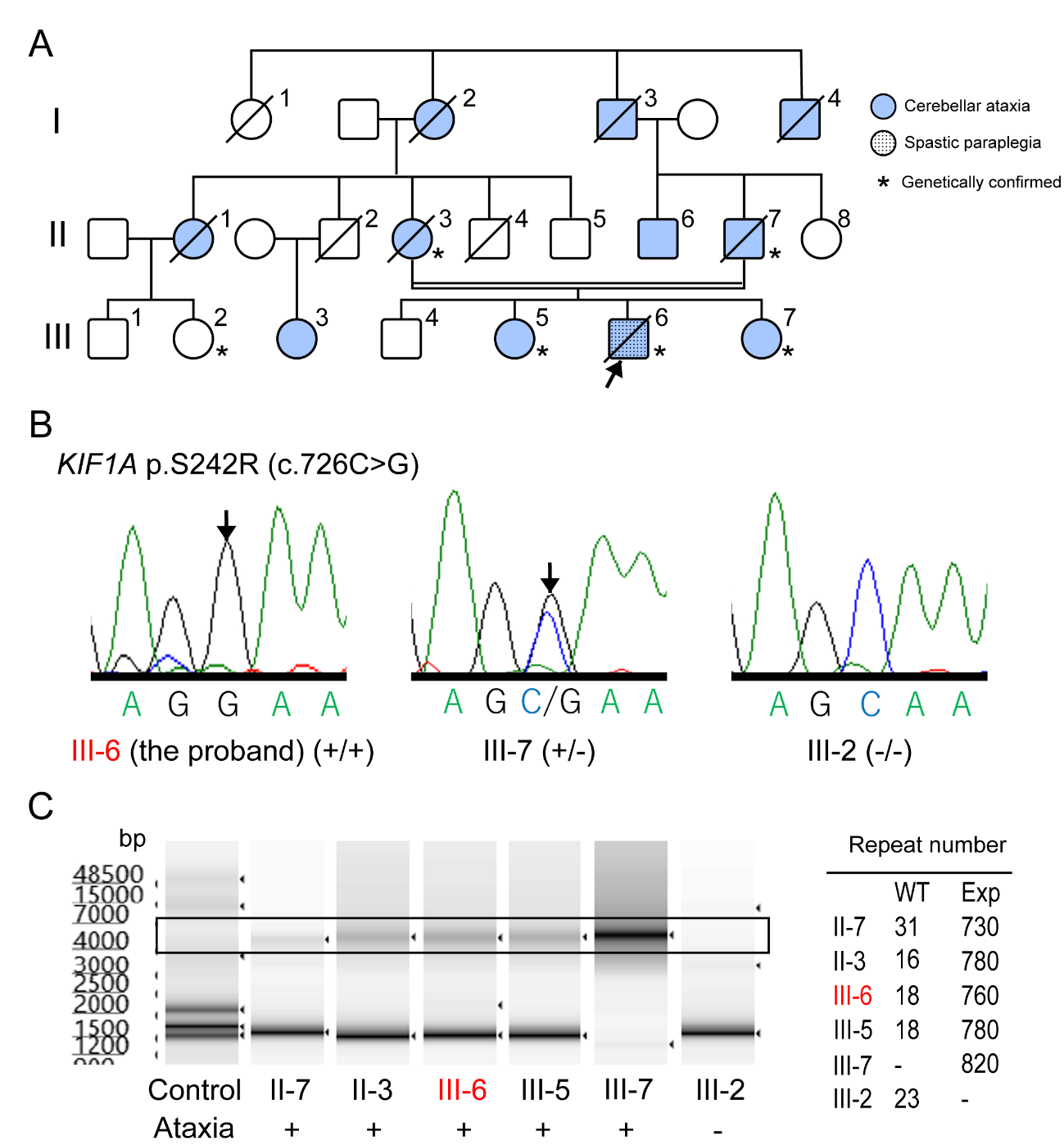


(**A**) Pedigree of this family. The proband (III-6, indicated by an arrow) was the only individual in the family exhibiting spastic paraplegia. (**B**) Sanger sequencing of the *KIF1A* variant. Black arrow indicates the variant. The missense p.S242R (c.726C>G) mutation was present in a homozygous state in III-6, while his parents (II-3 and II-7) and sister (III-7) were heterozygous carriers. The unaffected family member (III-2) tested negative for the mutation. (**C**) Abnormal ~2.7kb insertion between *BEAN* and *TK2*. The insertion was detected in a heterozygous state in II-7, II-3, III-6, and III-5, and in a homozygous state in III-7. Repeat number in the proband: 760 (pathogenic range 500–800) [5]. A and B are reproduced with permission from the *Journal of the Neurological Sciences* [4].

**Figure S2.** **Brain MRI and CT images.**

**
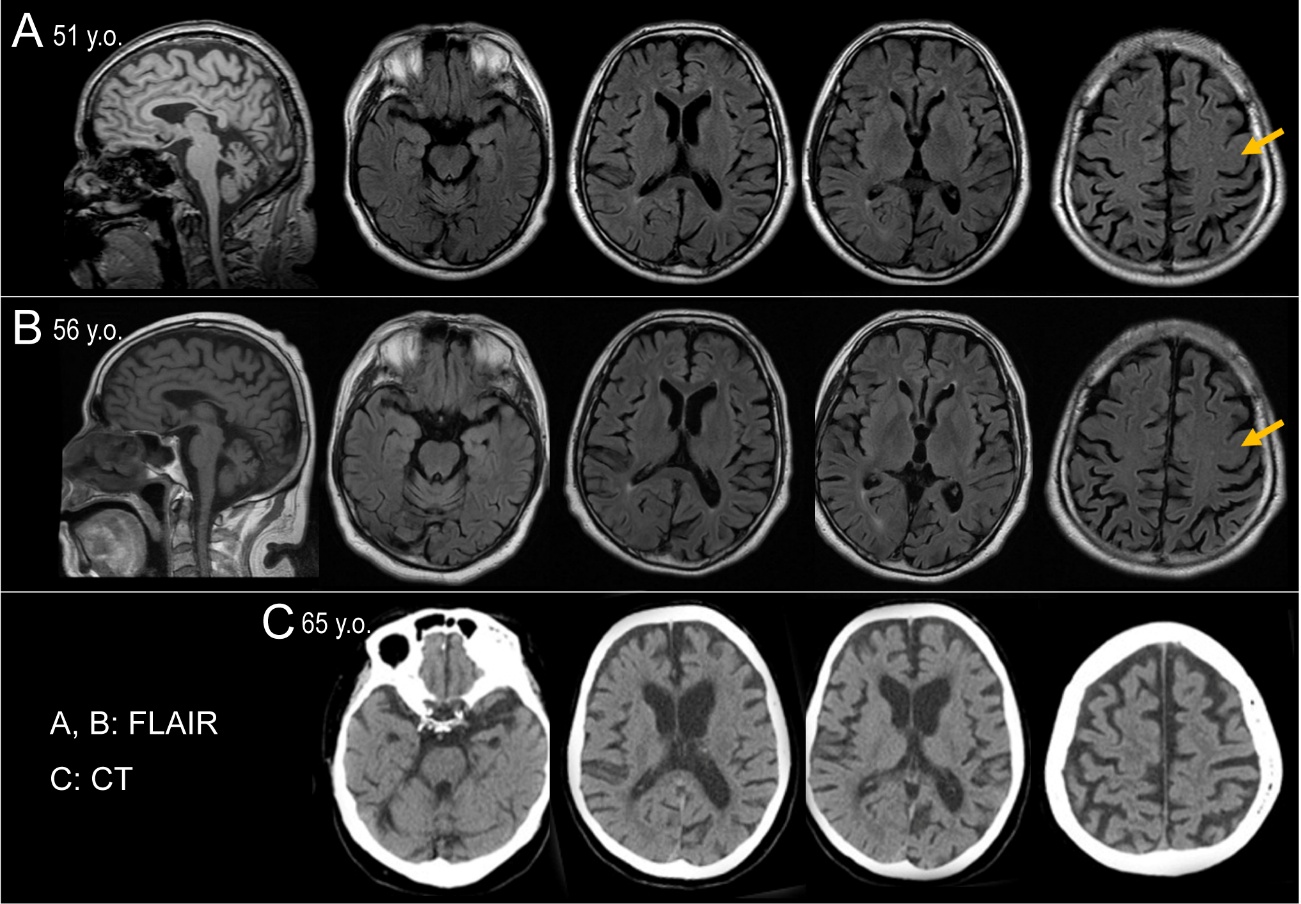
**

(**A**, **B**) Mild diffuse atrophy in the cerebrum and cerebellum, without prominent atrophy of the precentral gyrus (*arrows*). No appreciable progression was evident between the ages of 51 and 56. (**C**) Progressive atrophy of the frontotemporal lobes, including the precentral gyrus, with asymmetric medial temporal atrophy.

**Figure S3.** **p62 and phosphorylated TDP-43 (pTDP-43) immunoreactivity in the frontal lobe and corpus callosum.**

Abundant pTDP-43–positive NCIs; p62 shows granular positivity in a proportion of NCIs (*arrows*) and in GCIs.
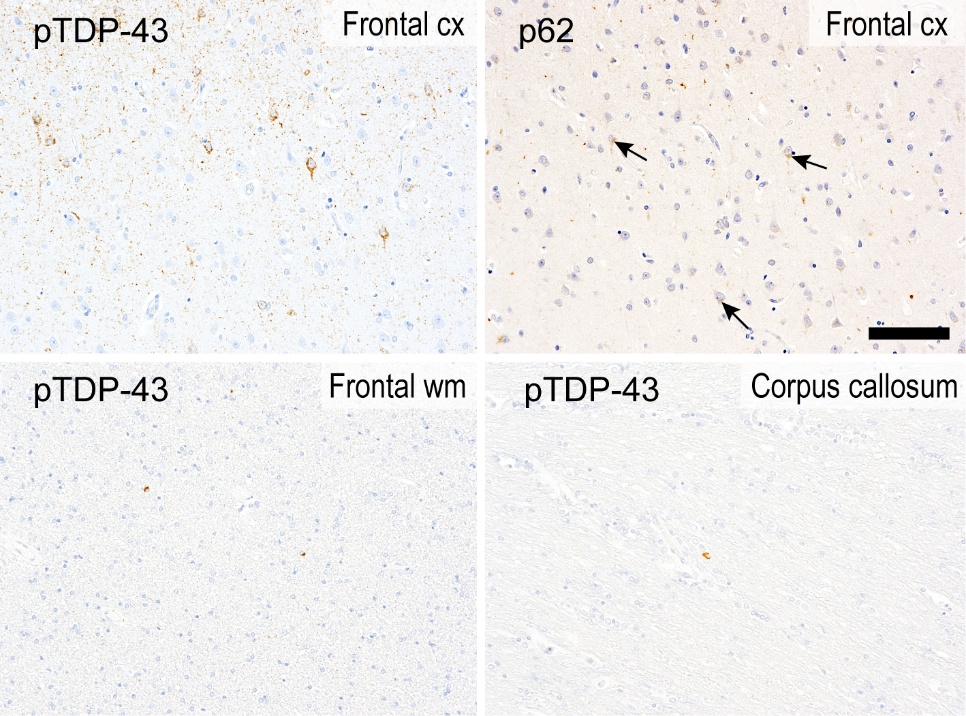
pTDP-43-immuno-reactive inclusions are rarely evident in the frontal white matter and corpus callosum. Bar = 100 μm.

**Figure S4. Pathological findings in the spinal roots and sural nerve.**

**
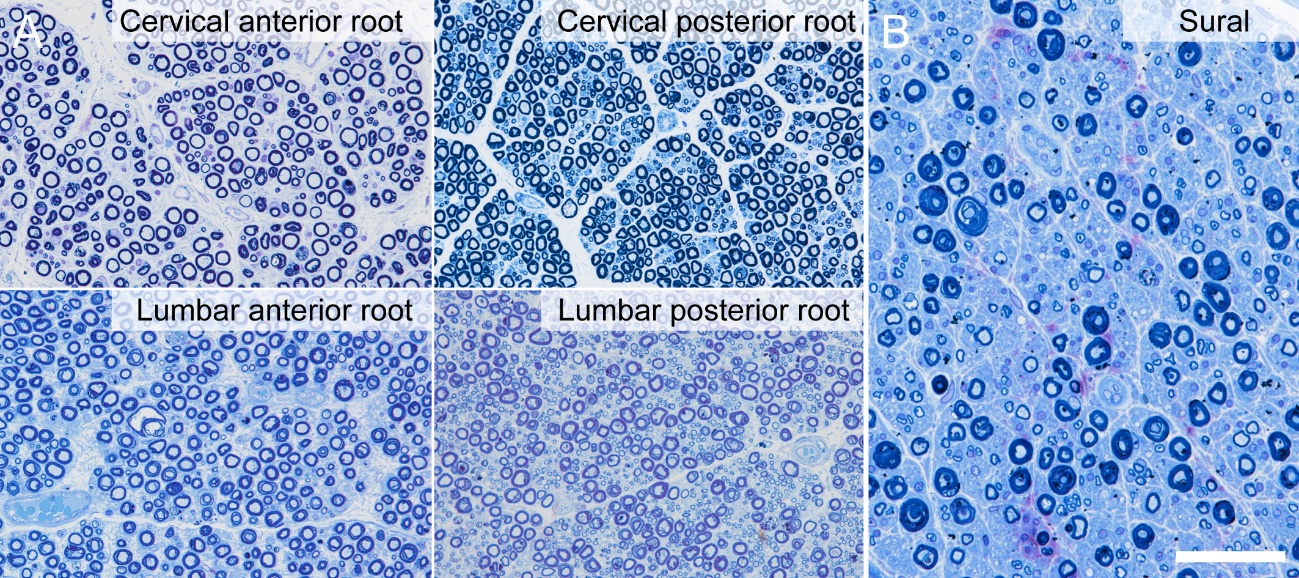
**

(**A**) The anterior and posterior spinal roots appeared thin on gross examination; however, the density of myelinated fibres was preserved. (**B**) In contrast, the sural nerve showed moderate loss of myelinated fibres, predominantly involving small-diameter fibres. Clinically, the patient had diabetes mellitus, with foot ulcers and markedly narrowed lower-limb arteries, findings that together suggest a substantial diabetic contribution to the sural nerve pathology. Toluidine blue staining. Bar in **B** = 50 μm for **A**; and 100 μm for **B**.

**Figure S5. Cerebellar pathology.**

**
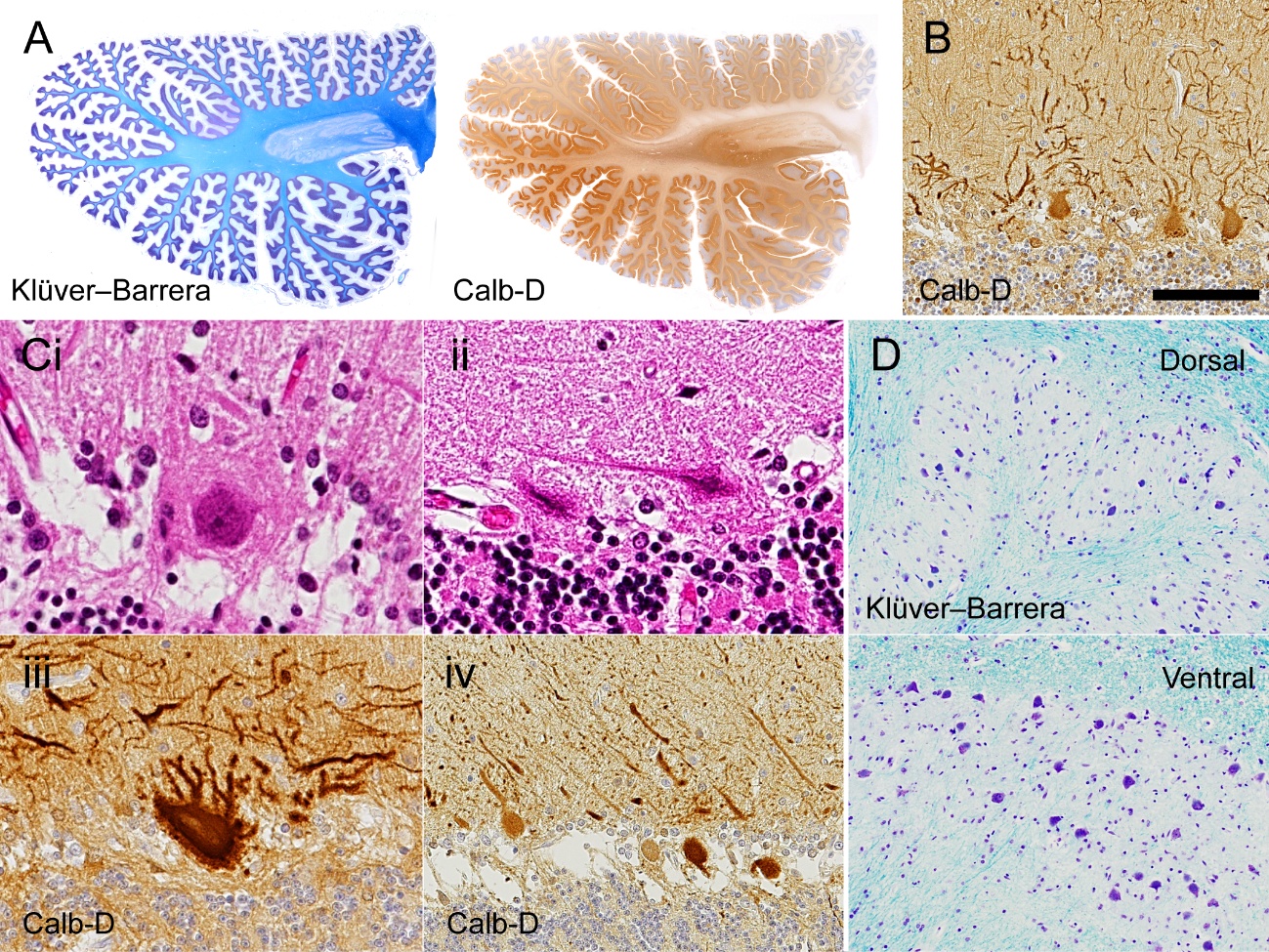
**

(**A**) Mild atrophy of the cerebellar cortex, with calbindin-D immunoreactivity largely retained in the lower part of the cortex. (**B**) Moderate loss of Purkinje cells (PJCs). (**C**) Higher-magnification views: (i) a Purkinje cell with halo-like amorphous material; (ii) nuclear atypia; (iii) cactus-like change; and (iv) decreased calbindin-D immunoreactivity. (**D**) Focal neuronal loss in the dorsal part of the inferior olivary nucleus. Calb-D, calbindin-D. Bar in **B** = 1.5 cm for **A**; 100 μm for **B**, **Civ**; 50 μm for **Ci**-**iii**; and 150 μm for **D**.

**Supplementary references**

1. Montine TJ, Phelps CH, Beach TG, et al. National Institute on Aging-Alzheimer’s association guidelines for the neuropathologic assessment of Alzheimer’s disease: a practical approach. ***Acta Neuropathol*** 2012;123:1-11.
2. McKeith IG, Boeve BF, Dickson DW, et al. Diagnosis and management of dementia with Lewy bodies: fourth consensus report of the DLB Consortium. ***Neurology*** 2017;89:88-100.
3. Saito Y, Ruberu NN, Sawabe M, et al. Staging of argyrophilic grains: an age-associated tauopathy. ***J Neuropathol Exp Neurol*** 2004;63:911-918.
4. Hasegawa A, Koike R, Koh K, et al. Co-existence of spastic paraplegia-30 with novel KIF1A mutation and spinocerebellar ataxia 31 with intronic expansion of BEAN and TK2 in a family. ***J Neurol Sci*** 2017;372:128-130.
5. Sato N, Amino T, Kobayashi K, et al. Spinocerebellar ataxia type 31 is associated with “inserted” penta-nucleotide repeats containing (TGGAA)n. ***Am J Hum Genet*** 2009;85:544-557.
6. Gijselinck I, Van Mossevelde S, van der Zee J, et al. C9orf72 intermediate repeats are associated with corticobasal degeneration, increased C9orf72 expression and disruption of autophagy. ***Acta Neuropathol*** 2019;138:795–811.
